# Evaluation of the anti-inflammatory, antioxidant and wound healing effects of pterostilbene in human gingival fibroblasts in vitro

**DOI:** 10.1007/s10266-024-01052-7

**Published:** 2025-01-21

**Authors:** Mukaddes Yerebakan, Gulay Tuter, Emin Umit Bagriacik, Nihan Oruklu, Tugce Guldurur

**Affiliations:** 1 Department of Periodontology, Faculty of Dentistry, Gazi University, Ankara, Turkey; 2 Department of Immunology, Faculty of Medicine, Gazi University, Ankara, Turkey

**Keywords:** Pterostilbene, Gingival fibroblast cells, Anti-inflammatory, Antioxidant, Wound healing

## Abstract

We aimed to investigate the wound-healing, antioxidant, and anti-inflammatory effects of pterostilbene (PTS) on human gingival fibroblasts (GF). Different concentrations of PTS were applied to GFs and cell viability was evaluated by MTT assay. GFs were stimulated by lipopolysaccharide (LPS) and the study groups were determined as LPS, LPS + 1 μM PTS, LPS + 10 μM PTS, and control. The most effective PTS concentrations were applied in a wound-healing model, with cell counts in the wound area assessed at 0, 24, 48, and 72 h. The effect of PTS on the release of interleukin-1β (IL-1β), tumor necrosis factor-α (TNF-α), interleukin-6 (IL-6), transforming growth factor-β (TGF-β), superoxide dismutase (SOD), glutathione peroxidase (GSH-Px), basic fibroblast growth factor (bFGF), and collagen type I (COL I) was assessed at 24 and 48 h by ELISA. The data was statistically analyzed. Our results showed that PTS had a dose-dependently negative effect on wound healing and cell proliferation at 10 μM concentration, but not at low concentration (1 μM). PTS exhibited a potent anti-inflammatory effect by reducing IL-6 and TNF-α levels, while also enhancing antioxidant activity, as evidenced by increased GSH-Px levels in the LPS + 1 μM PTS group (*P* < 0.05). According to our results, PTS could be a potential and promising substance with anti-inflammatory and antioxidant effects on LPS-stimulated GF. Therefore our results have merit in terms of providing pioneering data for future studies.

## Introductıon

Periodontal diseases are chronic inflammatory diseases that develop due to microbial dental plaque and its products, as well as the immune-inflammatory response initiated by the host against these products and various risk factors, resulting in the destruction of the supporting tissues of the teeth [1, 2]. According to studies conducted to explain the pathogenesis of periodontal disease, it is reported that the destruction in the periodontal disease process is affected by the long-term release of reactive oxygen species as well as the activation of the immune system [2–4].

Antioxidants are known to protect tissues from the destructive effects of inflammation and regulate the oxidative imbalance caused by inflammation [5]. Therefore, the homeostatic imbalance between reactive oxygen species and antioxidant defense systems plays a crucial role in the pathogenesis of periodontal diseases [6]. In recent years, the regulation of this balance by antioxidant molecules and their effects on various cell types have been subjects of research. For this reason, studies have intensified on the impact of antioxidants, which play a very important role in preventing oxidative damage, in the treatment of inflammatory diseases.

For example, Xiong et al. investigated the effects of quercetin, a natural antioxidant from the polyphenol group, on human gingival fibroblast cells stimulated with *P. gingivalis* lipopolysaccharide, and found that quercetin exhibited a dose-dependent anti-inflammatory effect [7]. In the study conducted by Li et al., the effects of resveratrol, another natural antioxidant, on gingival fibroblast cells stimulated with *P. gingivalis* LPS were investigated and according to the results of the study, strong anti-inflammatory and antioxidant effects were obtained [8]. In the study examining the effects of antioxidant ascorbic acid on the wound healing model created with gingival fibroblast cells, it was observed that ascorbic acid increased the expression of biomarkers related to wound healing activity [9]. In another in vitro study conducted by Nizam et al., it was determined that dose-dependent application of α-tocopherol, which has known antioxidant properties, accelerated the proliferation rate and wound healing process in periodontal ligament and gingival fibroblast cells [10].

There are studies in the literature examining the effects of various antioxidants on periodontal cells. However, the limited number of studies on the use of antioxidant natural molecules in the treatment of periodontal diseases, their healing effects on periodontal tissues, and their roles on periodontal cells indicate that further research on these antioxidants is needed. In line with this information, our study evaluated the effects of pterostilbene, a known potent antioxidant that has not previously been assessed for its effects in the field of periodontology, on gingival fibroblast cells.

Pterostilbene is a dimethylated analog of resveratrol, the most popular and widely studied of the stilbene group of antioxidants [11]. It acts as a phytoalexin, a compound that mainly functions to protect the plant against pathogens and other environmental stresses, and exhibits a range of pharmacological properties, notably anti-inflammatory, and antioxidant effects [12–14].

To the best of our knowledge, no studies have been encountered that investigate the effect of pterostilbene on periodontal diseases. Additionally, there is insufficient data regarding the potential cytotoxic and proliferative effects of pterostilbene on gingival fibroblasts. Therefore, we aimed to evaluate the anti-inflammatory, antioxidant, and wound-healing effects of various concentrations of pterostilbene on gingival fibroblast cells stimulated by lipopolysaccharide secreted by *Porphyromonas gingivalis (P. gingivalis)*. With this purpose, a wound model created on gingival fibroblast cultures was used to determine these effects of PTS and we aimed to analyze the levels of IL-1β, TNF-α, IL-6, TGF-β, SOD, GSH-Px, bFGF, and collagen type I.

### Materıals and methods

The present study was performed at the Gazi University Faculty of Medicine Immunology Laboratory. The study protocol was approved by Clinical Investigation Ethics Committee of Gazi University’s Faculty of Dentistry (ID E-21071282–050.99–462,173).

### Cell culture

Human primary gingival fibroblast cells were purchased from ATCC (ATCC® PCS-201–018, Virginia, USA). The cells were thawed at 37 °C. The cells were centrifuged fibroblast growth kit (ATCC® PCS-201–041, Virginia, USA) and precipitated by centrifugation at 1200 rpm for 5 min. The supernatant was then discarded and the cell pellet was resuspended with the recommended fibroblast growth kit in 25 cm2 culture flask. The cultures were incubated in a humidified incubator with 5% carbon dioxide at a temperature of 37 °C. Upon reaching 80% to 90% confluence, the cells were dissociated using trypsin–EDTA solution (%0.05 trypsin–EDTA with phenol red, Gibco) at 37 °C for 10 min, followed by the establishment of subcultures in culture plates. The amplification of a sufficient number of cells used for MTT, wound model, and ELISA stages was accomplished by repeating these procedural steps.

### Preparation of LPS and pterostilbene

LPS derived from *P. gingivalis* (InvivoGen US, LPS-PG Standard, San Diego, California) was purchased to induce an inflammatory response in gingival fibroblast cells. In our study, the most appropriate *P. gingivalis* LPS concentration was determined to be 1 μg/mL and LPS was prepared at a concentration of 1 μg/mL under the literatüre [7, 15–17]. 10 mg of pterostilbene (Phyproof® Reference Substances, trans-Pterostilbene, Vestenbergsgreuth, Germany) was prepared in 0.3 mL of dimethyl sulfoxide solution. Subsequently, to obtain a solution at the desired concentrations from the prepared stock solution, dilution was performed using the serial dilution method. Stock solution was diluted at 0.1 μM, 1 μM, 10 μM, 25 μM, 50 μM, 100 μM, 200 μM.

### Cell viability

Pterostilbene concentrations prepared at different doses were applied to the wells containing the cells. 96 cell plate was placed in the incubator for 1 day. Then, 10 μL of MTT solution (Carl Roth GmbH & Co. Kg, Thiazolyl Blue, Karlsruhe, Deutschland) was pipetted into control and cell wells, and the cells were further incubated at 37 °C for 4 h. After removal from the incubator, 100 μL of sodium dodecyl sulfate solution was added to the cells, and the 96-well plate was left to incubate again overnight. Gingival fibroblast cells proliferation and viability were performed at 24 h. Absorbance was measured at 570 nm in an ELISA reader.

### Preparation of in vitro wound-healing model

**Fig. 1:**
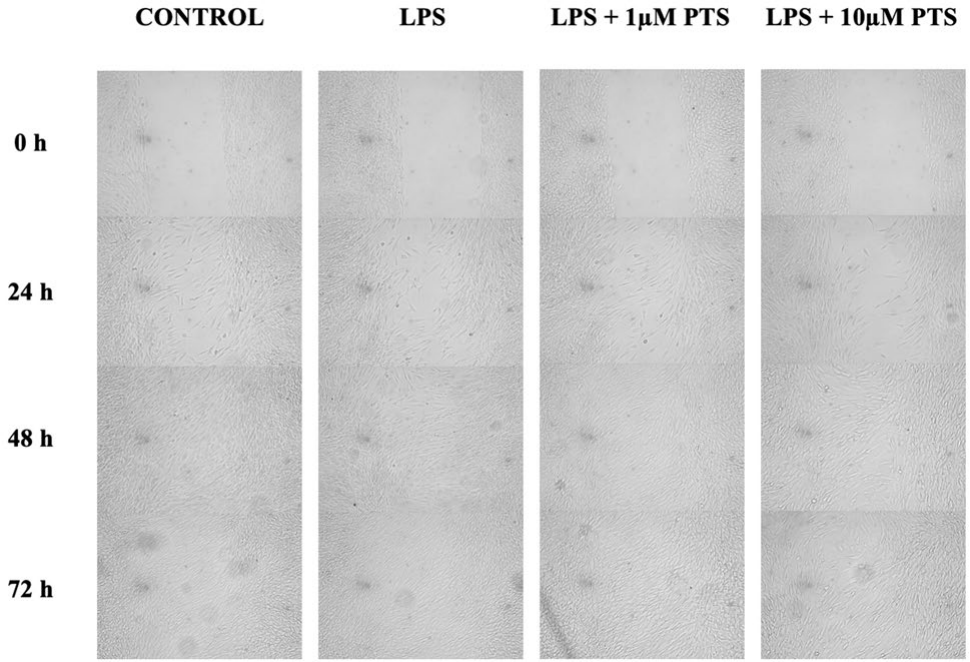
In vitro wound healing model images of human gingival fibroblast cells after 24, 48, and 72 h of incubation

A wound healing kit (Ibidi® Culture-Insert 2 Well in μ-Dish 35 mm, Gräfelfing, Germany) consisting of three parts designed for cell adhesion and proliferation was used. Silicone inserts, which included two rectangular wells separated by a barrier measuring 500 mm in width, were placed in cell culture dishes with a diameter of 35 mm. 70 μL of cell suspension, containing 10^4^ cells, was applied to each well of the silicone insert. The wound healing kit was then incubated at 37 °C in an environment with 5% CO2. Once the cells completely covered the wells, the silicone inserts were gently removed using sterile tweezers, creating a 500 μm wide cell-free area. The cell culture dishes were washed with phosphate-buffered saline and then filled with fresh medium for both control and test groups (control group, 1 μg/mL LPS, 1 μg/mL LPS + 1 μM PTS, 1 μg/mL LPS + 10 μM PTS). Images of the wound area were taken at 0, 24, 48, and 72 h under a fluorescence microscope (Leica DM4000) (Fig. 1). A transparent counting grid was used to count cells in standardized images. The cells in the wound area were counted and recorded, and the numerical data obtained were compared and evaluated as a percentage.

### Determination of ELISA

The concentrations of IL-1β, TNF-α, IL-6, TGF-β, SOD, GSH-Px, bFGF and Collagen Type I in supernatants were measured with enzyme-linked immunosorbent assay (ELISA) using human IL-1β (Elabscience® Human IL-1β ELISA kit, Houston, Texas, USA), TNF-α (Elabscience® Human TNF-α ELISA kit, Houston, Texas, USA), IL-6 (Elabscience® Human IL-6 ELISA kit, Houston, Texas, USA), TGF-β ((Elabscience® Human TGF-β ELISA kit, Houston, Texas, USA), SOD (Cayman Chemical Superoxide Dismutase Assay Kit, Ann Arbor, Michigan, USA), GSH-Px (Cayman Chemical Glutathione Peroxidase Assay Kit, Ann Arbor, Michigan, USA), bFGF (Elabscience® Human bFGF/FGF2 ELISA kit, Houston, Texas, USA) and Collagen Type I (Elabscience® Human COL1α1 ELISA kit, Houston, Texas, USA) ELISA kits.

### Statistical analyses

The statistical analysis was performed using the GraphPad Prism 9.5.1 software (GraphPad Prism Software Inc., San Diego, CA) program. Group comparisons were conducted using a one-way ANOVA, and differences between the two groups were assessed using Student's t-test. Statistical significance was determined when *P* < 0.05.

## Result

### Effect of pterostilbene on cell viability

**Fig. 2:**
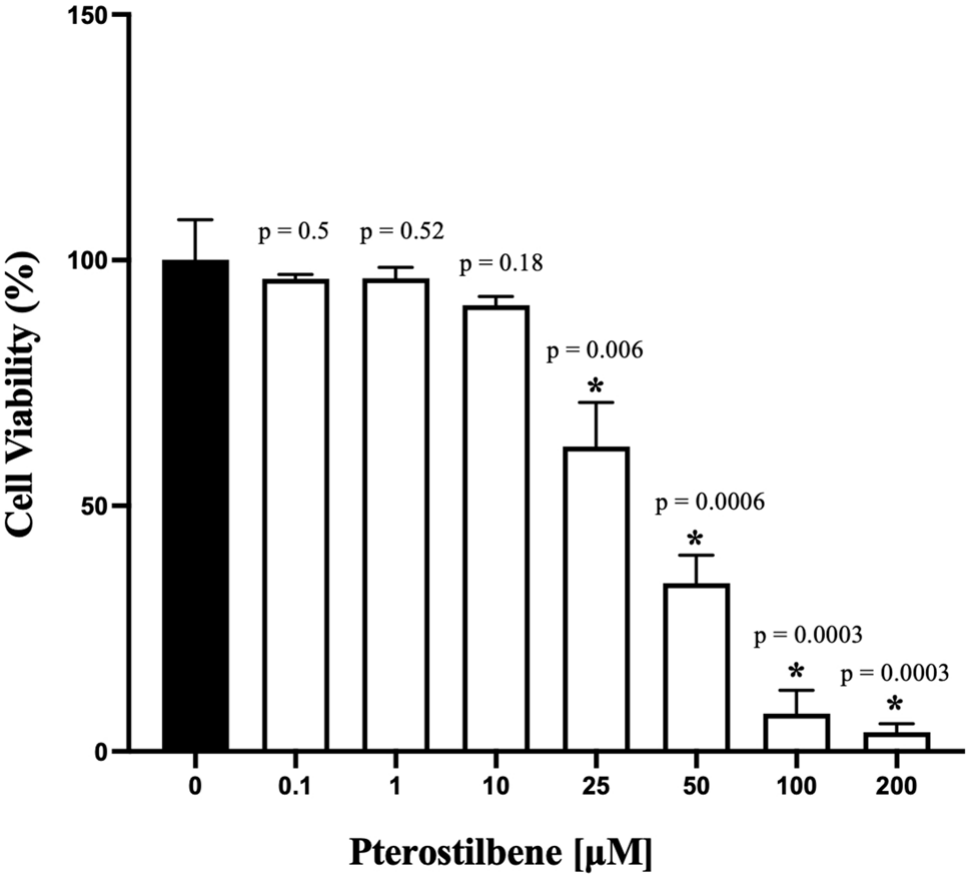
Evaluation of percent cell viability rates of gingival fibroblasts under different doses of pterostilbene applications at the 24th hour, Bars indicate ratios, error bars indicate standard deviation, *Statistical significance between the control group and other groups (*: *P* < 0.05)

According to the results obtained from the MTT assay at the 24-h mark, cell viability rates decreased significantly when compared to the control at Pterostilbene doses of 25 μM and higher. There was no statistically significant difference in cell viability rates at Pterostilbene doses of 10 μM and lower compared to the control (Fig. 2).

### Effect of Pterostilbene on a wound-healing model

**Fig. 3:**
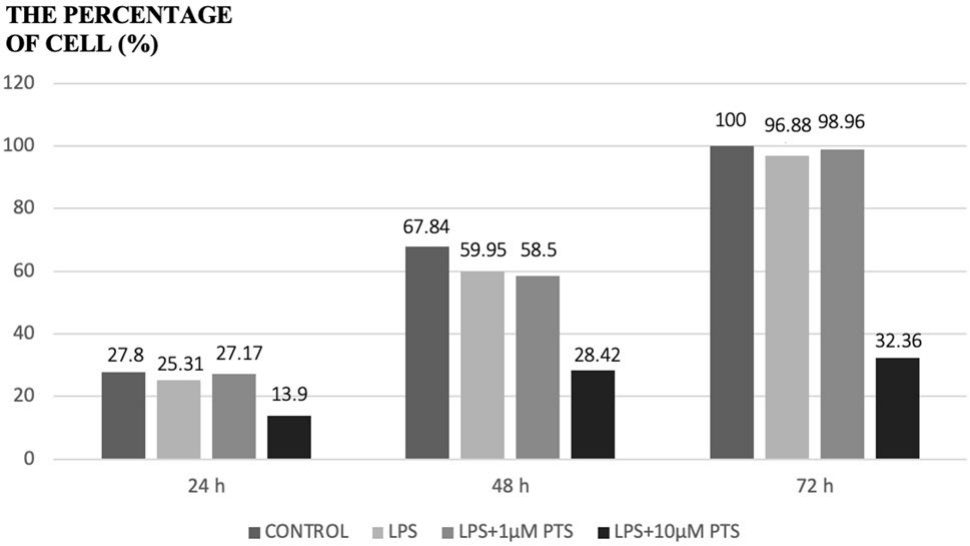
Gingival fibroblast cell percentages according to hours in the created wound model

At the start of the experiment (hour 0), the wound area did not contain any cells, hence the cell percentage was considered 0%. It was observed that the concentration of 1 μM pterostilbene had no significant impact on the proliferation of gingival fibroblast cells. However, it was noted that the concentration of 10 μM Pterostilbene significantly decreased cell proliferation when compared to other groups (Fig. 3).

### Measurement of IL-1β, TNF-α, IL-6, TGF-β, SOD, GSH-Px, bFGF and collagen type I production

**Fig. 4:**
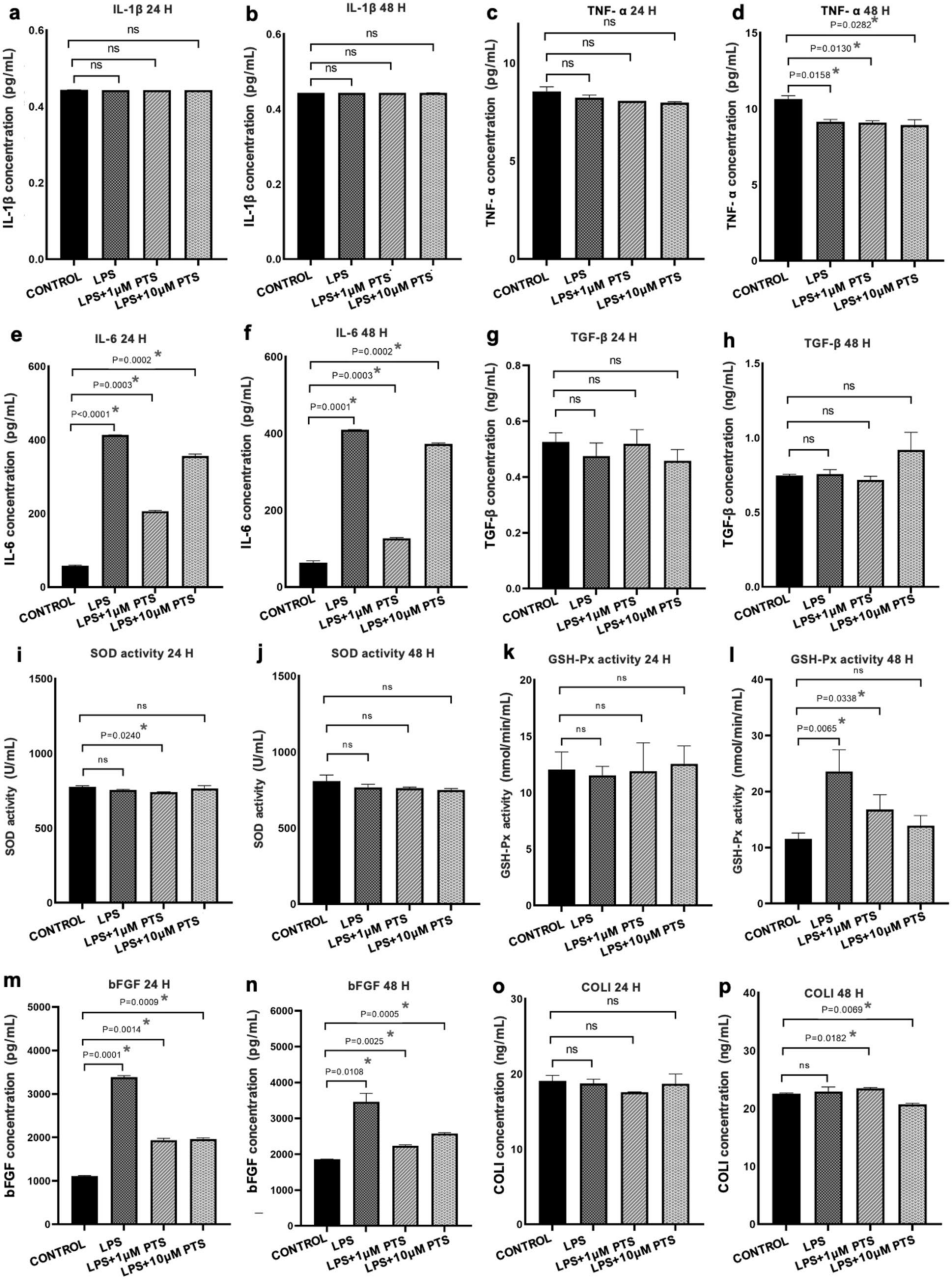
Evaluation of IL1-β, TNF-α, IL-6, TGF-β, SOD, GSH-Px, bFGF and COLI levels by ELISA method from the supernatants obtained from the cell culture medium of the groups at 24th and 48th hours. **a** 24th hour IL-1β ELISA findings. **b** 48th hour IL-1β ELISA findings. **c** 24th hour TNF-α ELISA findings. **d** 48th hour TNF-α ELISA findings. **e** 24th hour IL-6 ELISA findings. **f** 48th hour IL-6 ELISA findings. **g** 24th hour TGF-β ELISA findings. **h** 48th hour TGF-β ELISA findings. **i** 24th hour SOD ELISA findings. **j** 48th hour SOD ELISA findings. **k** 24th hour GSH-Px ELISA findings. **l** 48th hour GSH-Px ELISA findings. **m** 24th hour bFGF ELISA findings. **n** 48th hour bFGF ELISA findings. **o** 24th hour COLI ELISA findings. **p** 48th hour COLI ELISA findings. Bars indicate ratios, error bars indicate standard deviation, *Statistical significance between the control group and other groups (*: *P* < 0.05)

In our study, we determined that the ELISA kit used to evaluate IL-1β levels functioned properly. However, the IL-1β levels in both our control and experimental groups were found to be below the measurement range of the ELISA kit used (*P* > 0.05, Fig. 4a, Fig. 4b). After applying 1 μM and 10 μM pterostilbene, no statistically significant difference was observed in the TNF-α values obtained from supernatants collected at 24 h compared to the control group (*P* > 0.05, Fig. 4c). However, at 48 h, a statistically significant reduction in TNF-α levels was detected in all groups compared to the control group (Fig. 4d). The IL-6 levels of all experimental groups were found to be statistically significantly higher than the control group at both 24 and 48 h (*P* < 0.05, Fig. 4e, Fig. 4f). It was observed that the application of pterostilbene resulted in a decrease in IL-6 release at all time points compared to the LPS group. This reduction was found to be particularly pronounced in the LPS + 1 μM PTS group (Fig. 4e, Fig. 4f). There was no statistically significant difference in TGF-β levels obtained from supernatants collected at 24 and 48 h between control and experimental groups (*P* > 0.05, Fig. 4g, Fig. 4h). SOD levels obtained from supernatants at 24 h showed that the LPS + 1 μM PTS group significantly decreased compared to the control group (*P* = 0.0240, Fig. 4i). No statistically significant difference was detected for the other groups at 24 and 48 h (*P* > 0.05, Fig. 4i, Fig. 4j). GSH-Px levels obtained from supernatants collected at 24 h showed no statistically significant difference compared to the control group (*P* > 0.05, Fig. 4k). However, when GSH-Px levels at 48 h were examined, it was determined that the values for both LPS and LPS + 1 μM PTS significantly increased compared to the control group (*P* = 0.0065, *P* = 0.0338, Fig. 4l). In our study, it was found that bFGF levels significantly increased in all experimental groups compared to the control group at both 24 and 48 h. However, it was observed that the bFGF levels in both the LPS + 1 μM and LPS + 10 μM PTS groups were below those of the LPS group at 24 and 48 h (Fig. 4m, Fig. 4n). No statistically significant difference was observed in collagen type I levels at 24 h compared to the control group (*P* > 0.05). It was observed that collagen type I levels increased in both the LPS + 1 μM PTS and LPS + 10 μM PTS groups at 48 h compared to 24 h. This increase was only statistically significant in the 48-h LPS + 1 μM PTS group collagen type I levels compared to the control group (*P* = 0.0182, Fig. 4o, Fig. 4p).

## Dıscussıon

Studies in the literature have evaluated the antioxidant and anti-inflammatory properties of pterostilbene on various cell groups [13, 14]. While there are studies on the effects of pterostilbene on different cell groups, there is a lack of research on its impact on periodontal diseases. Insufficient data exists regarding the potential anti-inflammatory, antioxidant, and proliferative effects of pterostilbene on gingival fibroblasts.

In the first part of our study, we conducted an MTT test to assess the proliferative and cytotoxic effects of pterostilbene on gingival fibroblast cells. Due to the absence of a definitive concentration recommendation for pterostilbene on cells comprising the periodontium in the literature, we used a wide dose range for the MTT analysis, ranging from 0.1 to 200 µM. In our study, no statistically significant reduction in cell viability was observed at doses of 10 µM and below compared to the control group, and a significant reduction was observed at doses of 25 µM and above. Therefore, the most effective doses for wound healing and ELISA tests have been determined to be 1 µM and 10 µM.

Many different wound model methods have been developed from the past to the present, and to date, many studies have evaluated the effects of various compounds on cells using the wound model method. Similar to these previous studies on different molecules and different cells, an in vitro wound model was created to evaluate the proliferative effect of 1 μM and 10 μM pterostilbene application. It was observed that pterostilbene applied at a concentration of 1 μM PTS to LPS-stimulated gingival fibroblast cells in the created wound model did not have any positive effect on cell migration and proliferation. Our study observed that increasing concentrations of pterostilbene (LPS + 10 μM) significantly reduced cell proliferation and migration at 24, 48, and 72 h. When comparing the data from the MTT assay and the wound model, it is believed that high doses of pterostilbene may even have negative effects on wound healing at high doses.

In the last step of our study, to examine the anti-inflammatory effects of pterostilbene on gingival fibroblast cells, IL-1β, TNF-α, IL-6 and TGF-β biomarkers were evaluated; to assess its antioxidant effects, SOD and GSH-Px biomarkers were examined; and to investigate its effects on wound healing, bFGF and COL1 markers were assessed.

IL-1β, a pro-inflammatory cytokine, is a crucial mediator of the inflammatory response and plays a significant role in the pathogenesis of many chronic inflammatory diseases [18]. Due to the absence of studies in the literature investigating the effect of pterostilbene on IL-1β release from LPS-stimulated gingival fibroblasts, we have been unable to compare our findings. However, previous studies have shown that pterostilbene can reduce the secretion of IL-1β by exerting anti-inflammatory effects on various cells [19–21]. Despite the ELISA kit used to evaluate IL-1β levels functioning properly in our study, the IL-1β levels in both control and experimental groups remained below the measurable range of the ELISA kit used. This result suggests that the IL-1β levels remained below the sensitivity threshold of the ELISA kit used.

TNF-α is another pro-inflammatory cytokine that plays an important role in the regulation of immune response and tissue destruction [22]. While there is no research on the effects of pterostilbene on TNF-α release from gingival fibroblasts in cell culture studies, existing studies have shown that pterostilbene can reduce TNF-α release by exhibiting anti-inflammatory effects on different cells [19–21]. Similar to the above studies, our study also found that TNF-α levels significantly decreased in both the LPS + 1 µM PTS and LPS + 10 µM PTS groups by the 48th hour compared to the control group. Unlike previous studies, we used *P. gingivalis* LPS to stimulate gingival fibroblasts and observed that TNF-α levels in the LPS group remained lower than in the control group at 24 h and 48 h. In an in vitro study, it was reported that macrophages stimulated with *P. gingivalis* LPS produced lower levels of TNF-α and PGE2 compared to those stimulated with standard LPS [23]. This suggests that gingival fibroblasts in our study may have exhibited a similar response. Additionally, one study suggested that the response to *P. gingivalis* LPS may vary depending on the specific strain used and the method of LPS preparation [24].

IL-6 is a critical cytokine with multiple functions involved in regulating the host response during bacterial infection [25, 26]. Several studies in the literature have shown that pterostilbene has anti-inflammatory effects on various cells by reducing the release of IL-6 [19–21]. Lin et al. have shown that pterostilbene suppresses the production of proinflammatory cytokines such as IL-6, TNF-α, and IL-1β, as well as NO by inhibiting NF-κB in both in vitro and in vivo studies [19]. Similarly, in the study conducted by Liu et al., it was noted that the application of pterostilbene reduced inflammatory mediators such as TNF-α, IL-1β, and IL-6 in co-cultured hippocampal neuronal HT22 and astroglioma U251 cells [20]. In another in vitro study conducted by Lim et al., it was observed that the application of pterostilbene to RAW 264.7 cells stimulated with the periodontal pathogen Fusobacterium nucleatum resulted in a decrease in the expression of IL-1β, IL-6, and TNF-α [21]. Our study is the first to investigate the impact of pterostilbene on the release of IL-6 from human gingival fibroblast cells. At the same time, the results we obtained are consistent with previous in vitro studies [19–21]. In line with our findings, pterostilbene decreases the release of IL-6 from LPS-stimulated gingival fibroblasts with a dose of 1 μM PTS, suggesting its anti-inflammatory activity. Our data demonstrates that the reduction in IL-6 levels was less significant in the group treated with 10 μM pterostilbene compared to the group treated with 1 μM pterostilbene. This result can be interpreted to mean that increasing the dose of pterostilbene may have adverse anti-inflammatory effects on gingival fibroblasts.

TGF-β is a growth factor that can be produced by all cells in the body and serves as a cytokine with multiple functions [27]. The literature has reported in vitro and in vivo studies showing that pterostilbene can reduce TGF-β release by exhibiting anti-inflammatory effects [28, 29]. However, in our study, the TGF-β values obtained from the supernatants collected at 24 and 48 h after applying 1 μM and 10 μM pterostilbene to LPS-stimulated gingival fibroblast cells did not show a statistically significant difference compared to the control group.

Superoxide Dismutase is an enzyme that plays a crucial role in the antioxidant defense mechanism by protecting cells against the harmful effects of reactive oxygen species [30]. In our research, when we evaluated the SOD levels obtained from the supernatants collected at 24 and 48 h after applying 1 μM and 10 μM pterostilbene, we found that only the SOD levels at 24 h in the LPS + 1 μM PTS group were significantly decreased compared to the control group. Upon evaluating our results, we concluded that the application of pterostilbene did not have a significant effect on the SOD activity of gingival fibroblasts. In contrast to our study, previous in vitro studies on various cells have indicated that pterostilbene actually increases SOD activity [31, 32]. These variations in results may be attributed to differences in cellular responses to the materials used under in vitro and in vivo conditions.

GSH-Px protects cells from oxidative damage caused by hydrogen peroxide radicals by preventing the formation of hydroxyl radicals [33]. When the existing studies in the literature were examined, it was not possible to compare our findings due to the lack of studies investigating the effect of pterostilbene on GSH-Px release from gingival fibroblasts. However, other studies have reported that pterostilbene exhibits antioxidant effects on various cells and increases GSH-Px release [31, 32]. In the in vitro study conducted by Yang et al., which reported that pterostilbene prevents heart failure by reducing oxidative stress and inflammation, it was found that the application of pterostilbene to RAW264.7 and H9C2 cells increased the expression of glutathione peroxidase, superoxide dismutase, and catalase [31]. Similarly, the in vitro study conducted by Ullah et al. evaluated the effects of pterostilbene on mouse preimplantation embryo cells. The study showed that the application of pterostilbene significantly increased the expression of glutathione peroxidase, SOD, catalase, and heme oxygenase-1 through Nrf2 nuclear translocation [32]. We observed no significant difference in GSH-Px levels obtained from the supernatants after 24 h of pterostilbene application compared to the control group, but at 48 h, GSH-Px levels significantly increased in the group treated with 1 μM pterostilbene. This finding aligns with previous in vitro studies confirming the antioxidant capacity of pterostilbene. The result we obtained is significant in demonstrating, that pterostilbene increases GSH-Px levels with the application of a 1 μM dose, thereby showcasing its antioxidant capacity.

bFGF induces and speeds up the growth of most of the major cells involved in wound healing, such as fibroblasts [34]. Due to the presence of studies in the literature showing that natural antioxidants increase bFGF release in gingival fibroblasts. Our study evaluated the role of pterostilbene, a natural antioxidant, on bFGF release from gingival fibroblasts [9, 10]. The results of our study showed that bFGF levels increased in all groups compared to the control group at both the 24th and 48th hours, and this increase was statistically significant. The higher levels of bFGF in both the LPS + 1 μM PTS and LPS + 10 μM PTS groups compared to the control group suggest that pterostilbene may increase bFGF release. This result is similar to the findings of previous in vitro studies conducted with other antioxidants. However, the bFGF levels in both pterostilbene treatment groups at both doses were lower than the bFGF levels in the LPS group. This situation suggesting that pterostilbene application may cause a change in the cellular response.

Collagen type I is considered the primary component of the extracellular matrix in the periodontium and plays a crucial role in remodeling periodontal tissues [35]. Studies have shown that natural antioxidants can enhance the release of collagen type I in gingival fibroblasts. Therefore, we chose to use pterostilbene, a natural antioxidant, in our study [9, 10]. Our findings revealed a significant increase in collagen type I levels in the group treated with LPS + 1 μM at 48 h compared to the control group. This result is in line with the results of previous in vitro studies with other antioxidants (α-tocopherol, ascorbic acid) [9, 10]. Additionally, we observed a rise in collagen type I levels in the LPS + 1 μM PTS group at 48 h compared to 24 h, indicating a potential ongoing effect of pterostilbene on collagen production and potential benefits for wound healing. However, our results also showed that collagen type I levels were lower in the group treated with 10 μM pterostilbene compared to the control group at 48 h, suggesting that higher doses of pterostilbene may have adverse effects on cellular response.

## Conclusıons

In our study, the dose-dependent negative effect of pterostilbene on cell proliferation in wound healing revealed that it may have a cytotoxic effect at high concentrations (10 μM), but it does not have an anti-proliferative effect at low concentrations (1 μM). For this reason, we believe that studies using different dose ranges at PTS are needed to determine the appropriate doses of pterostilbene in vitro studies related to periodontal wound healing and regeneration. According to our findings, a strong anti-inflammatory effect resulting in a decrease in IL-6 and TNF-α levels and a strong antioxidant activity resulting in an increase in GSH-Px levels were detected with the application of 1 μM pterostilbene to LPS-stimulated gingival fibroblasts. Our findings provide hope that pterostilbene can be used in the treatment of periodontal disease at doses that have been proven to be appropriate and non-cytotoxic. In addition, our results are important in terms of providing pioneering data for in vitro and experimental animal studies on the subject. Further, in vivo studies are needed to confirm the findings.

## Data Availability

All data supporting the findings of this study are available within the article and its supplementary information files.
